# Neurologic Manifestations of Autoimmune Diseases

**DOI:** 10.1155/2012/683212

**Published:** 2012-12-11

**Authors:** Simone Appenzeller, Yehuda Shoenfeld, Jozélio Freire de Carvalho

**Affiliations:** ^1^Rheumatology Division, Department of Medicine, State University of Campinas, 13083-887 Campinas, SP, Brazil; ^2^Zabludowicz Center for Autoimmune Diseases, Sheba Medical Center-Sackler Faculty of Medicine, Tel Aviv University, 52621 Tel Aviv, Israel; ^3^Rheumatology Division, University Hospital, Federal University of Bahia, 41810-080 Salvador, BA, Brazil

Autoimmune diseases have a broad spectrum of clinical manifestations. Among them, neurologic involvement, both from the central nervous system as well as from the peripheral nervous system, is among the most challenging manifestations, regarding diagnosis and treatment [1]. Neurologic involvement in systemic lupus erythematosus and antiphospholipid syndrome is frequently observed, and reported and several etiological factors are involved including immunomediated damage and thrombosis [1, 2]. In this issue, we focused on less frequently described neurologic manifestations. 

One of the papers describes complex regional pain syndrome (CRPS) in a patient with adult onset Still's disease. CRPS is a chronic neuropathic pain disorder characterized by neuropathic pain and associated with local edema and changes suggestive of autonomic involvement such as altered sweating, skin color, and skin temperature of the affected region. Although it has been described in patients following trauma, psychiatric conditions, and malignancy, the association of CRPS with other autoimmune diseases is rare. 

The paper entitled “*Sensory neuronopathy and autoimmune diseases*” describes the importance of recognizing this clinical entity, frequently characterized by ataxia and often associated with systemic autoimmune diseases. The authors discuss not only the epidemiologicy and pathophysiology of this syndrome, but also emphasize clinical findings and discuss specific autoimmune diseases where sensory neuronopathy is frequently observed. 

Broad aspects of the involvement of the nervous system in Sjogren's disease are described in the paper entitled “*Neurological disorders in primary Sjögren's syndrome*.” The authors not only discuss the clinical aspects and physiopathology of central and peripheral nervous system involvement, but also emphasize biological markers for neurologic involvement in this disease. In the practical aspect of the paper, the authors discuss treatment, including immunosupressive drugs, anti-TNF drugs, and other biological therapies such as rituximab. 

Localized scleroderma is a rare disease, characterized by sclerotic lesions. A variety of presentations have been described, with different clinical characteristics and specific prognosis. Once considered an exclusive cutaneous disorder, the neurologic involvement present in localized scleroderma has been described in several case reports. Seizures are most frequently observed, but focal neurologic deficits, movement disorders, trigeminal neuralgia, and mimics of hemiplegic migraines have been reported. In the paper “*Neurologic involvement in scleroderma en coup de sabre*” the authors describe clinical and radiologic aspects of neurologic involvement in localized scleroderma. Although no randomized controlled trials exist for treatment of neurologic manifestations in scleroderma en coup de sabre, the authors describe the current literature findings. 

In the paper “*Significant changes in the levels of secreted cytokines in brains of experimental antiphospholipid syndrome mice*” the authors examined the role of proinflammatory and anti-inflammatory cytokines in experimental APS (eAPS) mice brains. The authors showed that other immune mediators, apart from autoantibodies, are important in the inflammatory and degenerative processes in the APS brain. These results are encouraging and endeavor clinical studies in APS patients with neurologic symptoms with immunomodulatory drugs. 

*Simone Appenzeller* *Yehuda  Shoenfeld* *Jozélio Freire de Carvalho*
